# Implicit Processes, Self-Regulation, and Interventions for Behavior Change

**DOI:** 10.3389/fpsyg.2017.00346

**Published:** 2017-03-08

**Authors:** Tom St Quinton, Julie A. Brunton

**Affiliations:** ^1^Department of Sport, Health and Nutrition, Leeds Trinity UniversityLeeds, UK; ^2^School of Biomedical Sciences, University of LeedsLeeds, UK

**Keywords:** self-regulation, implicit, conscious, non-conscious, dual-process

## Abstract

The ability to regulate and subsequently change behavior is influenced by both reflective and implicit processes. Traditional theories have focused on conscious processes by highlighting the beliefs and intentions that influence decision making. However, their success in changing behavior has been modest with a gap between intention and behavior apparent. Dual-process models have been recently applied to health psychology; with numerous models incorporating implicit processes that influence behavior as well as the more common conscious processes. Such implicit processes are theorized to govern behavior non-consciously. The article provides a commentary on motivational and volitional processes and how interventions have combined to attempt an increase in positive health behaviors. Following this, non-conscious processes are discussed in terms of their theoretical underpinning. The article will then highlight how these processes have been measured and will then discuss the different ways that the non-conscious and conscious may interact. The development of interventions manipulating both processes may well prove crucial in successfully altering behavior.

## Conscious Processes in Behavior Change

The dominant theories within health psychology aim to predict and explain behavior in order to guide the construction of interventions (Michie et al., 2009). A theoretical understanding is important, particularly as interventions based on theory are more likely to be effective (Michie and Johnston, 2012). With a focus on conscious, reflective processes, these cognitive models introspect attitudes, risk perceptions, and beliefs (Bandura, 1989; Ajzen, 1991; Schwarzer, 2008), typically through self-report. Such processes are theorized to encompass motivation which subsequently leads to the development of a behavioral intention. Thus, intention mediates the effect of motivation on behavior. Although studies have found that expectancy-value models, such as the Theory of Planned Behavior (TPB: Ajzen, 1985), predict intention well (McEachan et al., 2011), there is still a large proportion in behavior left unexplained (Armitage and Conner, 2001). Unfortunately, the use of correlational data, which insufficiently validates a theory (Weinstein, 2007), far outweighs the number of studies attempting to change these processes through the development of interventions (Hardeman et al., 2002). This is despite the prevalence of clear methodological guidelines on belief alteration (Ajzen, 1991). Of those that have done so, however, successful motivational manipulations have not resulted in actual change (Sniehotta, 2009), thus suggesting people are generally motivated but struggle to transfer these positive intentions into action (Chatzisarantis and Hagger, 2005). Theoretical and methodological issues have been offered to explain the intention-behavior gap such as intention stability, lack of volitional control, and poor study design (Sheeran and Abraham, 2003; Ajzen, 2015). There are different explanations as to why individual’s fail at completing their previously stated intentions. Such explanations include forgetting, distraction, conflict, procrastination, action initiation, and control (Orbell et al., 1997; Webb and Sheeran, 2006; Gollwitzer, 2015). Crucially, such reasons go beyond processes of motivation.

## Self-Regulation and Planning

The transfer of motivation into action is dependent heavily on one’s ability to self-regulate. Self-regulation is the ability to overcome obstacles, get back on track, and ward off distractions from tempting stimuli (Baumeister et al., 1994). Thus, possessing such regulatory skills is crucial in fostering the enactment of a previously stated intention. Proceeding the motivational phase of behavior, action implementation occurs during the volitional phase of striving. As such, self-regulation is important during the phase of volition, with its purpose to facilitate positive motivational states. Goal-setting techniques and raising an individual’s level of self-efficacy are ways to regulate conscious processes. For example, a high belief in behavioral achievement will determine how one recovers from set-backs and thus decreases the chances of becoming derailed (Bandura, 1989). One of the widely used strategies to facilitate self-regulation involves planning (Schwarzer, 2008). One type of planning is implementation intentions (Gollwitzer, 1999), which consists of precise descriptions of critical situations in which to perform a target behavior in terms of ‘if/then’ specifications (Gollwitzer, 1999). Such plans create a strong affiliation between a situational cue and the goal-directed behavior, so that this planned behavior may be triggered and initiated automatically when the cue signaling the specified situation is encountered. It is argued that intentions do not directly induce actions, but that they lead to highly specific plans, which in turn prompt action initiation (Gollwitzer, 1999). A similar strategy is action planning which is often erroneously conflated with implementation intentions, but includes more detail in the planning content (Hagger and Luszczynska, 2014). Included within the post-intentional stage of the Health Action Process Approach (Schwarzer, 2008), action planning involves precise specifications of ‘when,’ ‘where,’ and ‘how’ behavior will be performed. In addition to action planning, coping plans can also be used to facilitate self-regulation by identifying potential inhibitors and barriers to behavior (Sniehotta et al., 2005). Although action and coping plans have convergent validity, it is best to treat them as two separate strategies (Schwarzer, 2016). More specifically, as one can only identify potential obstacles when the behavior is first imagined, action plans precede coping plans.

## Motivation and Volition

Due to the importance of motivational and volitional processes in changing behavior, researchers have combined the two within interventions. White et al. (2012) successfully increased physical activity within chronic disease sufferers by targeting identified motivational beliefs from the TPB alongside planning strategies. French et al. (2012) found increases in walking behavior in a combined TPB motivation and volition intervention compared to those targeting motivation and volition separately. Studies have also used planning strategies with other theories of motivation to successfully change behavior including the Protection Motivation Theory (e.g., Milne et al., 2002; Zhang and Cooke, 2012), and the decisional balance sheet (e.g., Prestwich et al., 2003).

Although such work has been useful, a gap between planning and behavior has also been identified (Sniehotta, 2009), thus suggesting the presence of other mediators and moderators. This gap has been addressed through the recent integration of preparatory behaviors (Barz et al., 2016) and action control (Fernández et al., 2016). Preparatory behaviors are those formed *a priori* to the behavior, thus act as a sub-goal (Barz et al., 2016). Action control refers to the monitoring and evaluation of action with respect to the behavior (Fernández et al., 2016). Preparatory behaviors, like planning strategies, are prospective routes to change; whereas action control involves concurrent strategies. In placing these two strategies as moderators, that is planning is facilitated by the identification of preparatory behaviors and implementation of action control, other moderators may also influence their success such as self-efficacy (Bandura, 1989) and cognitive resource depletion (Baumeister et al., 2007). For example, Barz et al. (2016) found preparatory behaviors to be more useful for those with less efficacy beliefs. That is, the transfer of positive intentions via planning and preparatory behaviors was more prevalent in those who didn’t believe in their abilities.

In summary, it can be suggested that motivation results in the formation of a behavioral intention, which can lead to the development of volitional strategies such as action and coping plans. The identification of preparatory behaviors alongside action control then facilitates the transfer of these plans into action. The inclusion of volitional strategies can therefore significantly assist self-regulation.

## Non-Conscious Processes

Aside from conscious processes and planning approaches, recent interest within health models of behavior change concerns the inclusion of implicit processes (Sheeran et al., 2013). Such processes operate at the non-conscious level without awareness, and are seen as being relatively automatic and fast. As such, it is argued that processes of which popular models of cognition such as the TPB target, are insufficient to explain and change behavior (Sniehotta et al., 2014), with the lack of explanatory power a resultant of processes such theories fail to take into consideration. The ability to self-regulate and thus enact behavior may be heavily dependent on implicit processes.

## Measuring the Implicit

Despite the ease of which reflective processes can be assessed using self-report measures, it is far more difficult to measure implicit processes since individuals may not be able to accurately reflect such processes (Hagger et al., 2015). Implicit processes have been typically measured using the Go/No-Go Association Task (Nosek and Banaji, 2001), affective priming task (Klauer and Musch, 2003), and the Implicit Association Test (IAT; Greenwald et al., 1998). Such measures prevent socially desirable responses often found in self-report. Concerning the IAT, which is probably the most common, participants are presented with a set of stimulus on a computer which they then classify into one of two categories through the use of a key press. The response latencies are used to calculate implicit preferences. One problem with this measure is that it requires the use of opposite stimuli and there is no obvious dichotomy for certain behaviors, such as physical activity (Rebar et al., 2015). Moreover, it is unclear whether the test reveals a preference for a stimulus over another or a dislike for a stimulus over the alternative (Blanton et al., 2006). For example, does an individual prefer healthy foods more than unhealthy foods, or is their distaste for unhealthy foods more prevalent than their love for healthy foods. The Single-Category Implicit Association (Karpinski and Steinman, 2006), which only has one target category, has been developed to address this problem. Other concerns refer to the specificity of implicit measures and its internal validity. The TPB assesses reflective beliefs specified at the target, action, context, and time (TACT), whereas non-conscious measures appear to provide assessments on a broader level (Perugini and Richetin, 2011). Furthermore, response latencies may be a product of physical ability; not cognitive processes (Jaccard and Blanton, 2006).

## Dual Process Theories

The relationship between implicit and explicit processes can be framed using a variety of dual-processing models. On the whole, these models describe one level of processing that is deliberate and requires reflection, and another level of processing that is implicit, automatic and requires minimal cognitive resources. However, these definitions vary and have been mixed depending on the theoretical stance taken. For example, according to Hollands et al. (2016), non-conscious behavior can be ascribed to situations when the causal link between a stimulus and the behavior is not recognized, thus implying consciousness is not automatic and is under individual control. This sense of authorship may be illusionary (Wegner, 2002), however, as a strong subjective sense of a causal role isn’t sufficient to attribute causation (Bargh, 2008). In terms of the models, just like the plethora of change theories identifying reflective processes, a similar pattern has emerged integrating both types of processes. More specifically, these theories offer differing interactions between the processes, resulting in a confused understanding of the two (Payne and Gawronski, 2010). Understanding how the two processes interact and the type of interventions required will be discussed later.

## Intervention Implications of Non-Conscious Processes

It is important to identify the role that the non-conscious plays in behavior change and self-regulation as this has important implications for intervention design. Papies (2016a) suggests that cues do not affect behavior directly, but do so by activating cognitive structures that have been formed by previous experiences in similar situations. Thus, there is a mediation of implicit processes, with the activation of implicit structures or schemata significant. This poses the question of how difficult such processes are to change as a consequence of prior learning and experiences (Wilson et al., 2000). If features of the situation can be changed so that they alter which of a person’s associations stored in memory becomes activated (Strack and Deutsch, 2004), this would provide avenues for target. Similarly, if such measures represent implicit processes responding to the environment, these external influencers can be used to target behavior via these processes. Of note here are prior situated conceptualizations and their interactions with environmental stimuli. If the environment is producing change wholly, the individual could be viewed as a blank state, ‘tabula rasa’ known to the behaviorists. An implicit change would, in turn, produce similar responses in each individual (Payne and Gawronski, 2010). Thus, there is a tension between whether processes are representative of the individual or their environment (Payne and Gawronski, 2010). Due to the processes generated from prior experiences, such a conclusion is unlikely. What is more likely is the activation of implicit processes, resulting in implicit processes that are still amendable to change (Gawronski and Bodenhausen, 2006). As the mere perception of objects activates stored memory associations (Hall and Fong, 2007), priming can be used to manipulate desired responses (Bargh et al., 2012). Such priming influences have been discussed recently by Papies (2016b). Planning can also be used to prime positive behavior. That is, when the situation is encountered, the cue enacts the behavior automatically (Gollwitzer and Brandstätter, 1997).

In addition to focusing on *what* is automatically activated through external affects, attention can be placed on *how* people respond to the stimuli. Such a process parallels the procedures involved in reflective models such as Cognitive Behavioral Therapy (CBT) whereby responses to automatic conscious processes bear the focus of intervention (Ellis, 1957; Beck, 1964). Changing how an individual responds to implicit processes can be done in a number of ways such as evaluative conditioning (Levey and Martin, 1975), approach/avoidance training (AAT; Chen and Bargh, 1999), Attention Bias Modification Treatment (ABMT; MacLeod et al., 2002), and mindfulness (Segal et al., 2002). Following the ideas of classical conditioning (Pavlov, 1927), evaluative conditioning refers to changes in liking or disliking that are due to the pairing of stimuli (De Houwer, 2007). AAT involves multiple trials wherein participants are taught to overcome automatic action tendencies by learning to approach one class of stimuli and avoid its counterpart. ABMT weakens attentional responses by substituting the anticipated stimulus with a neutral one (Sheeran et al., 2013). Within mindfulness, attention shifts from the content of thoughts and feelings to the experience encountered within the present moment (Baer et al., 2006). Thus, in comparison to the challenging and realignment of maladaptive thoughts apparent within CBT, the aim of mindfulness is a refocus of awareness, which decreases the influence of implicit processes. Responses to unwanted influences can also be controlled using plans (Gollwitzer et al., 2011). Furthermore, coping plans can be used to prevent the urge of implicit processes overriding conscious processes by intervening between responses. Specifically, precise descriptions of coping mechanisms automatically activate and trigger responses to decrease the unhelpful behaviors’ influence. In all of these approaches, the individual regains a sense of volition over implicit processes rather than succumbing to the activated schemata.

## When the Conscious/Non-Conscious Do Battle

In addition to the influence of implicit processes, reflective processes also have a role to play in behavior. The development of the reflective system, which is seen as evolutionary new, performs certain tasks that the non-conscious cannot (Baumeister and Bargh, 2014). As stated earlier, the exact relation between the processes varies within each dual model. The following are potential interactions and their impact on behavior.

Both processes can influence behavior independently or act as moderators (i.e., the influence of the non-conscious is moderated by consciousness or the influence of conscious processes is moderated by the non-conscious) (Rebar et al., 2016). The limited space allocated for consciousness is facilitated by the implicit processes triggered automatically whilst providing adaptive responses to the environment (Baumeister and Bargh, 2014). Reflective processes may develop an intention and, once repeatedly linked with a stimulus, become under the guidance of the non-conscious through automatic activation (Gardner, 2015). Thus, once activated, action is induced through non-conscious processes instead of prior reflective states (Gardner, 2015). Targeting intention and habits may therefore be fruitful (Allom et al., 2016). Automatic processing may be the default mode, guiding behavior in accordance with one’s implicit attitudes unless an individual is prompt into deliberation (Evans and Stanovich, 2013). When motivation and opportunity is high, reflective processes may be in control compared to the automated influences when such motives and opportunity are low (Fazio, 1990). Due to its finite resource and susceptibility to depletion, a weakened cognitive state could hand self-control over to non-conscious processes despite having motivation and opportunity (Friese et al., 2009). Engaging in persistent decision making, complex experiences, and constant restraints is likely to drain the battery of self-control (Baumeister et al., 2007), leading to mental contamination (Wilson and Brekke, 1994). Tempting, unhealthy hedonic outcomes may be in contrast with the reflective, healthier outcomes (Strack and Deutsch, 2004). Affective attitudes, distinct from instrumental processes, could be seen as implicit routes to behavior (Conner et al., 2011). Such affective beliefs have been found to better predict short-term choices compared to instrumental beliefs governing long-term outcomes (Morris et al., 2016). Thus, prior positive long-term motivational intentions developed from the careful consideration of behavioral pros and cons could be overridden in the wake of affective outcomes. Finally, in utilizing a neurobiological approach, the influence of intentions on behavior can be moderated by executive function and behavioral prepotency (Hall and Fong, 2015). Those possessing higher inhibitory ability are more likely to suppress and inhibit urges and thus enable control to remain in the reflective system.

Although their relation is manifold, what is clear is the stronger the influence of the non-conscious, the more one has to consciously overcome it (Bargh, 2014). The tug-of-war between the processes, encapsulated well within the horse rider metaphor (Friese et al., 2011), determines whether the reflective or implicit prevails (Hofmann et al., 2008). The strength of the competing schemas resulting from *boundary conditions* will determine the ‘winner’ (Strack and Deutsch, 2004). Although the concordance between implicit and explicit processes in health psychology remains unclear and some assert that reflective processes still account for implicit factors through mediation (e.g., Jaccard and Blanton, 2006; Ajzen and Dasgupta, 2015), research has moved from simply acknowledging non-conscious existence to examining their interactions and the situations that influence different responses (Hagger, 2016).

## Conclusion

Although research in behavior change has primarily concerned conscious processes, it is important to account for implicit processes in addition to its more common reflective counterpart. Such influences offer an explanation as to why there appears to be a significant gap between what one intends to do and what is actually performed, thus taps into problems of self-regulation. Despite the inclusion of implicit processes in several theories of behavior change, there remains a lack of effective interventions (Hollands et al., 2016). Due to the rapid progression in establishing a science of behavior change (Michie et al., 2008), linking techniques with determinants of change (Michie and Abraham, 2004) and establishing which combine best to facilitate change (Michie et al., 2009) would prove valuable. The number of techniques required could be of particular use (Dombrowski et al., 2012); specifically as different techniques are needed to change the different processes. Although there are concerns surrounding intervention delivery and reporting (Michie and Prestwich, 2010; Dombrowski et al., 2016), interventions and techniques should be included within the taxonomies currently dominated by reflective processes (Sheeran et al., 2013). Such additions may satisfy recent criticisms of popular reflective theories (e.g., Sniehotta et al., 2014).

## Author Contributions

The article was written by the first author, TSQ. The second author, JB, provided guidance, drafting and final approval.

## Conflict of Interest Statement

The authors declare that the research was conducted in the absence of any commercial or financial relationships that could be construed as a potential conflict of interest.
